# Protecting Against Sun Exposure: Which SPF Will You Recommend?

**DOI:** 10.6004/jadpro.2017.8.2.1

**Published:** 2017-03-01

**Authors:** Pamela Hallquist Viale

Cutaneous melanoma is a major cause of mortality and morbidity in the United States, and in younger adults, the disease is the second most common invasive cancer after breast cancer (Reed et al., 2012). Although recent approval of new therapies assists greatly in the treatment of melanoma, reducing the incidence of this skin cancer is critical.

We know that solar ultraviolet radiation is a cause of melanoma and that sun exposure plays a role in the development of additional skin cancers such as basal cell and squamous cell carcinomas. We’ve also seen an alarming trend of increasing new cases of this daunting disease in women, connected with the use of indoor tanning booths.

Although we counsel patients and the public on the use of sunscreen and sun-protective clothing, we sometimes stumble when asked the correct sun protective factor (SPF) to use for adequate sun protection. The SPF numbers of available preparations vary widely, and the numbers often depend on the source of the recommendation. We know that SPF lotion is essential in everyday use, and we know that frequent application and reapplication after exposure to water is critical to reduce sun damage. But what SPF is best to provide protection against development of skin cancers and melanomas? Is it necessary to use an SPF of 75 or 100? Or is daily use of SPF 20 enough to provide protection?

## SUN PROTECTION FACTOR AND MELANOMA RISK

There is little quality research published on the above question, but in a just-published study by Ghiasvand and colleagues, researchers sought the answer to sunscreen use, SPF, and subsequent melanoma risk (Ghiasvand, Weiderpass, Green, Lund, & Veierod, 2016). In Norway, the incidence of melanoma has risen sharply in the past 10 years to become the highest in the world (Ghiasvand et al., 2016). The prospective study was based on a Norwegian population of 143,844 women aged 40 to 75 years at inclusion, with 1,532,247 person-years of follow-up and 722 cases of melanoma. Using a multivariable Cox proportional hazards regression, the researchers estimated the association between sunscreen use (never, SPF < 15, and SPF > 15) and melanoma risk.

The study results revealed that the users of sunscreen experienced significantly more sunburns as well as vacations where sunbathing occurred and were more likely to be users of indoor tanning booths. Use of a sunscreen with SPF > 15 was associated with a statistically significant decrease in the risk of developing melanoma compared with use of a sunscreen with a lower SPF (< 15). The estimated decrease in melanoma with the general use of the higher SPF sunscreen preparations in the study population was 30% (Ghiasvand et al., 2016).

## PREVIOUS RESEARCH IN SKIN CANCER AND SUN PROTECTION

The Ghiasvand study was specific to melanoma, but it is notable as the first trial to examine the SPF factor necessary to show a reduction in the risk of development of the disease in a large cohort study. A previous randomized, controlled trial by Green et al. studied the regular use of sunscreen (1,621 participants receiving either daily or discretionary sunscreen application in combination with beta carotene or placebo; Green, Williams, Logan, & Strutton, 2011). The results demonstrated that 10 years after trial cessation, 11 new melanomas had been identified in the daily sunscreen group vs. 22 in the discretionary group, representing a reduced rate of disease. The daily participants used a sunscreen containing an SPF of 16% (Green et al., 2011). However, it is important to note that this trial was conducted in Australia, an environment with increased solar radiation and a heightened awareness of the potential for skin cancer associated with sun exposure.

A previous study by van der Pols and colleagues reported on the use of regular sunscreen in reducing squamous cell skin cancer in another Australian population (van der Pols, Williams, Pandeya, Logan, & Green, 2006). Interestingly, although the regular use of sunscreen (SPF not specified) reduced the incidence of squamous cell carcinoma by almost 40%, the incidence of basal cell carcinomas was not significantly different from those randomized to daily sunscreen use compared with the participants not applying daily sun protection.

## IMPLICATIONS FOR ADVANCED PRACTITIONERS

Advanced practitioners are often in the position of recommending sun protection in the form of clothing and shade. We also recommend the use of sun-protection lotion, but with the myriad of choices in SPF, we may be unsure exactly which product and SPF to use for maximum protection. With the recently published study by Ghiasvand and colleagues, we can reasonably suggest the use of a product with at least an SPF of 15 or greater. Importantly, Ghiasvand and colleagues estimate that based on the results of the study, the use of a product with an SPF > 15 by all women aged 40 to 75 in Norway could potentially reduce the incidence of melanoma in this population by 18%. This is a marked decline of a disease that has an increasing incidence and yet can be reduced in a measure under our direct control. Future research will continue to refine the best approaches to reduce the incidence of this challenging disease.
